# Mediastinal Lymphadenopathy Mimicking Malignancy on Positron Emission Tomography: A Case of Anthracotic Lymphadenitis Associated With Biomass Fuel Exposure

**DOI:** 10.7759/cureus.114669

**Published:** 2026-08-17

**Authors:** Julyan Al-Fori, Maryam Amer Malik, Dinakar Unnithan, Moyna Dwyer, Amer Saleem

**Affiliations:** 1 Respiratory Medicine, Milton Keynes University Hospitals NHS Foundation Trust, Milton Keynes, GBR; 2 Medicine, The Aga Khan University, Karachi, PAK; 3 Radiology, Milton Keynes University Hospitals NHS Foundation Trust, Milton Keynes, GBR; 4 Pathology, Milton Keynes University Hospitals NHS Foundation Trust, Milton Keynes, GBR

**Keywords:** anthracotic lymphadenitis, biomass fuel exposure, ebus-tbna, fdg-avid pet-ct, mediastinal lymphadenopathy, mediastinoscopy, pigment-laden macrophages

## Abstract

Fluorodeoxyglucose positron emission tomography computed tomography (FDG PET-CT) is an essential, highly sensitive modality for evaluating suspected thoracic malignancies. However, high FDG avidity merely reflects increased glucose metabolism and is not specific for cancer, frequently leading to false-positive findings in benign inflammatory and granulomatous conditions. Here, we report a case of a 65-year-old woman with intense PET-avid mediastinal and hilar lymphadenopathy and a partially calcified right upper lobe lesion, raising strong suspicion for thoracic malignancy or reactivation of tuberculosis. Moreover, a detailed environmental history revealed over four decades of exposure to biomass fuel smoke. Additionally, endobronchial ultrasound-guided transbronchial needle aspiration (EBUS-TBNA) yielded abundant pigment-laden macrophages without granulomas or malignant cells. Radiological suspicion for malignancy remained high, and diagnostic uncertainty persisted, prompting surgical mediastinoscopy, which definitively confirmed anthracotic lymphadenitis. This case underscores the clinical importance of obtaining a comprehensive environmental exposure history, particularly regarding biomass fuel smoke, when evaluating FDG-avid mediastinal disease. It highlights the major diagnostic limitations of PET-CT in differentiating benign inflammatory conditions from malignancy and reinforces the necessity of histological confirmation prior to initiating invasive treatment or assuming a cancer diagnosis.

## Introduction

Fluorodeoxyglucose positron emission tomography computed tomography (FDG PET-CT) is an essential, highly sensitive modality for the staging, restaging, and evaluation of suspected thoracic malignancies. However, increased 18F-FDG accumulation is non-specific and reflects heightened intracellular glucose metabolism rather than malignant transformation alone. FDG-PET identifies tissues with increased glucose utilization rather than detecting malignant cells specifically. Activated inflammatory cells, including macrophages and lymphocytes, can also demonstrate increased glucose metabolism and therefore increased FDG uptake, potentially mimicking malignant disease on PET imaging [1-3]. Inflammatory states characterized by activated neutrophils, lymphocytes, and tissue macrophages can demonstrate marked metabolic activity, resulting in high standardized uptake values (SUVs) that closely mimic nodal metastasis or lymphoma. Beyond active granulomatous infections such as tuberculosis and fungal diseases, benign non-infectious conditions can produce intense FDG avidity in mediastinal and hilar lymph nodes [1-4].

Among these underrecognized benign causes is anthracotic lymphadenitis, a condition driven by the accumulation of carbonaceous and inorganic microparticles within nodal histiocytes. Globally, prolonged exposure to biomass smoke and environmental air pollutants are major contributors to particulate deposition in the tracheobronchial tree and regional lymphatics. Biomass fuel exposure refers to repeated inhalation of smoke generated by burning organic fuels such as wood, charcoal, crop residues, or animal dung, particularly when used for cooking or heating in poorly ventilated indoor environments. These retained insoluble particles induce a persistent, low-grade inflammatory response and sustained macrophage activation, frequently yielding high uptake on PET imaging despite only modest physical enlargement of the affected lymph nodes [5-7].

Differentiating anthracotic lymphadenitis from malignant involvement or reactive infectious processes (such as reactivation tuberculosis) presents a significant clinical challenge, particularly in patients from endemic regions or those with relevant environmental risk factors. While minimally invasive sampling via endobronchial ultrasound-guided transbronchial needle aspiration (EBUS-TBNA) is the first-line investigation for mediastinal nodal staging, cytological findings of pigment-laden macrophages without distinct granulomas or malignant cells may leave diagnostic uncertainty when radiological suspicion remains high. In such discordant scenarios, surgical tissue confirmation via mediastinoscopy remains critical to establish a definitive diagnosis and prevent inappropriate disease overstaging or unnecessary oncological therapies [5-7].

## Case presentation

A 65-year-old woman was referred for assessment of mediastinal and hilar lymphadenopathy, with mild shortness of breath on exertion. She reported no associated red flag symptoms, including weight loss or hemoptysis. Her past medical history included type 2 diabetes mellitus, diabetic retinopathy, and gastritis.

Her past medical history included type 2 diabetes mellitus, diabetic retinopathy, and gastritis. She was a non-smoker, and she had resided in Afghanistan until 2011, followed by approximately five years in Pakistan. Notably, she reported over four decades of significant exposure to biomass fuels including wood, charcoal, and animal dung used for indoor cooking associated with poorly ventilated settings.

**Figure 1:**
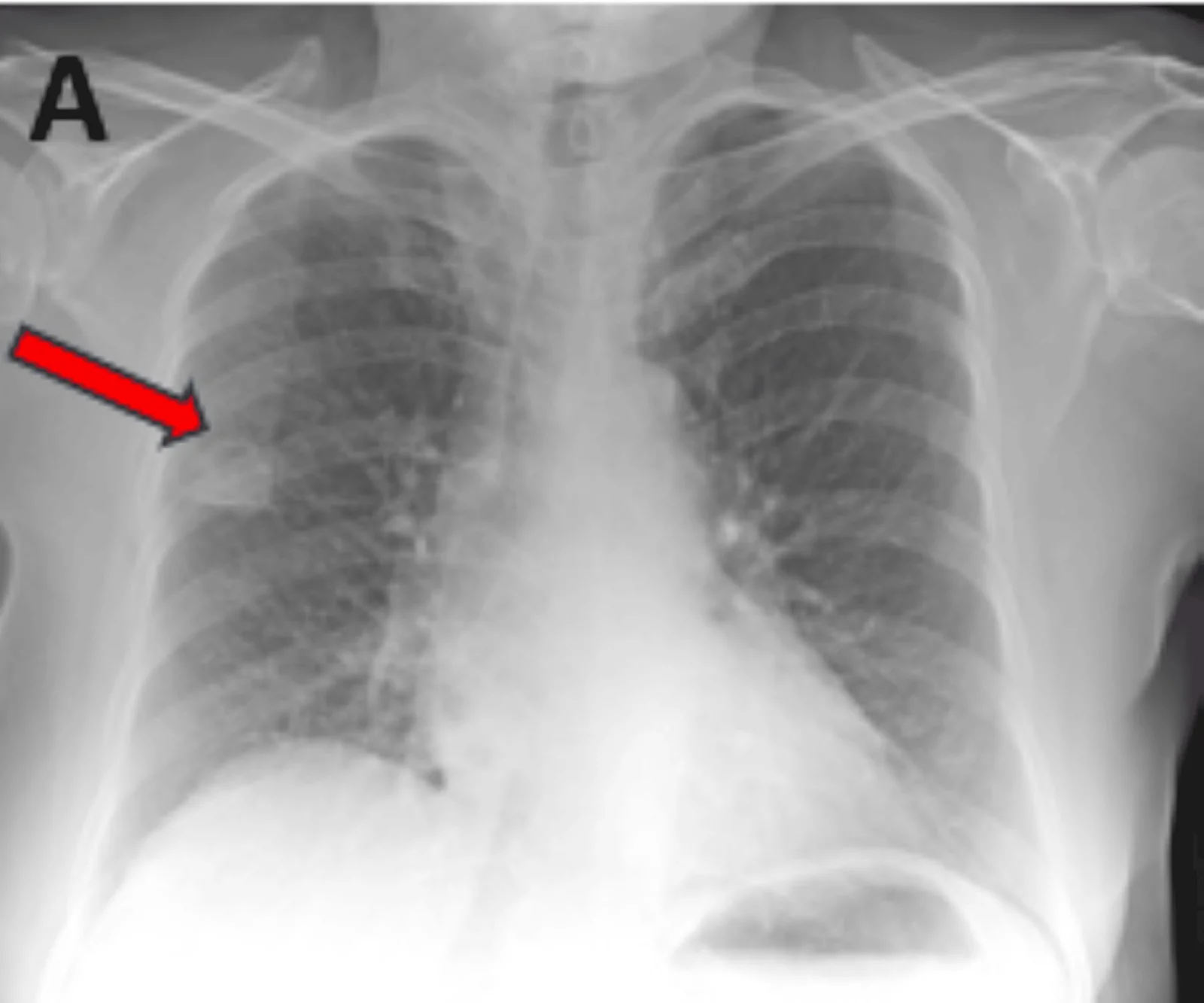
Right upper lobe opacity (red arrow)

**Figure 2:**
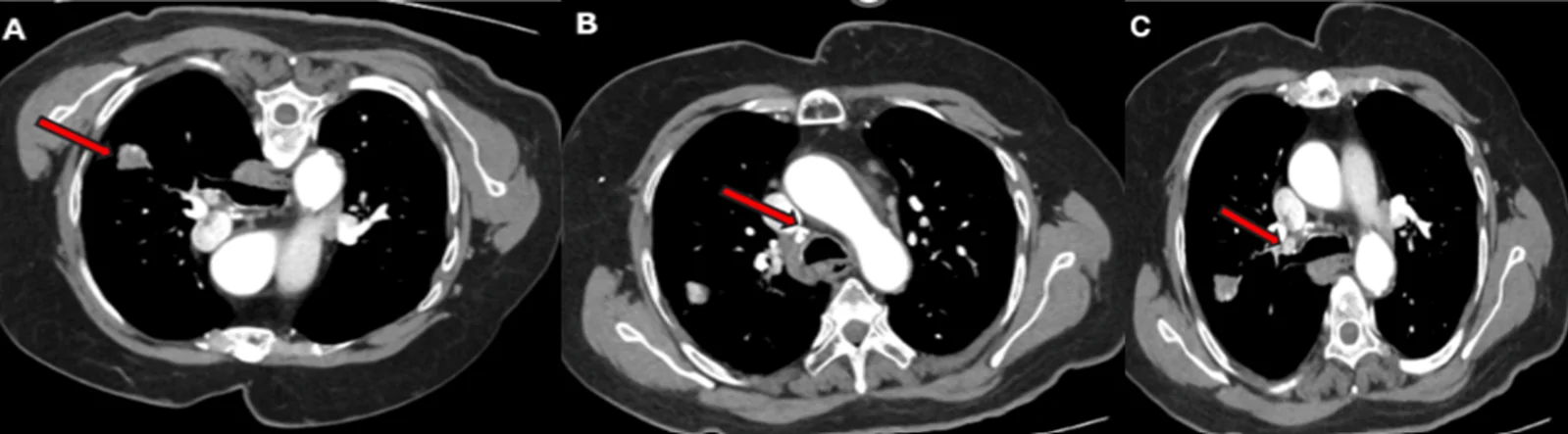
Heterogenous partially calcified mass within the basal segment of the right upper lobe (A), enlarged 4R with calcification (B), and enlarged hilar node (C)

**Figure 3:**
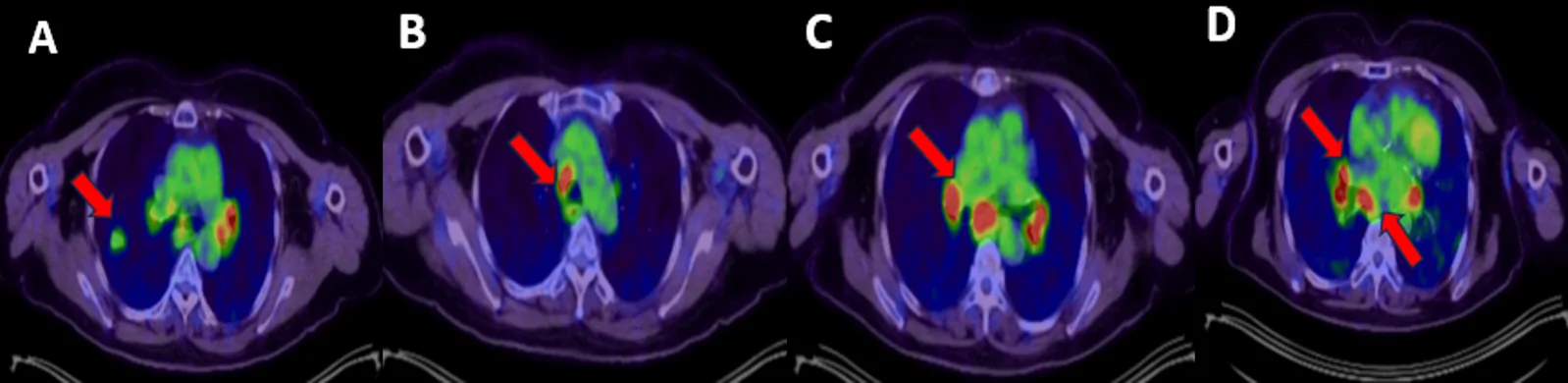
Faint uptake (SUVmax: 2.3) within a part-calcified 18 mm right upper lobe nodule (A) and increased metabolic uptake in mediastinal and hilar lymph nodes (B-D) SUVmax: maximum standardized uptake value

Initial evaluation included a chest radiograph demonstrating a right upper zone opacity (Figure 1) and an interferon-gamma release assay (IGRA), which was positive. Contrast-enhanced computed tomography (CT) of the thorax revealed a partially calcified right upper lobe lesion measuring 2.1 × 2.0 cm (Figure 2A) along with enlarged right hilar and mediastinal lymph nodes. Additionally, a calcified station 4R node was also noted (Figure 2B, 2C). Subsequent positron emission tomography computed tomography (PET-CT) demonstrated intense FDG uptake within the mediastinal and hilar lymph nodes with only faint uptake in the partially calcified right upper lobe lesion (Figure 3A-3C).

Given these radiological findings, primary concerns were strongly about thoracic malignancy or reactivation of tuberculosis. Thus, endobronchial ultrasound-guided transbronchial needle aspiration (EBUS-TBNA) of the mediastinal lymph nodes demonstrated numerous CD68-positive pigment-laden macrophages with no evidence of granulomas or malignant cells. Moreover, microbiological investigations, including acid-fast bacilli smear and culture, were both negative.

**Figure 4:**
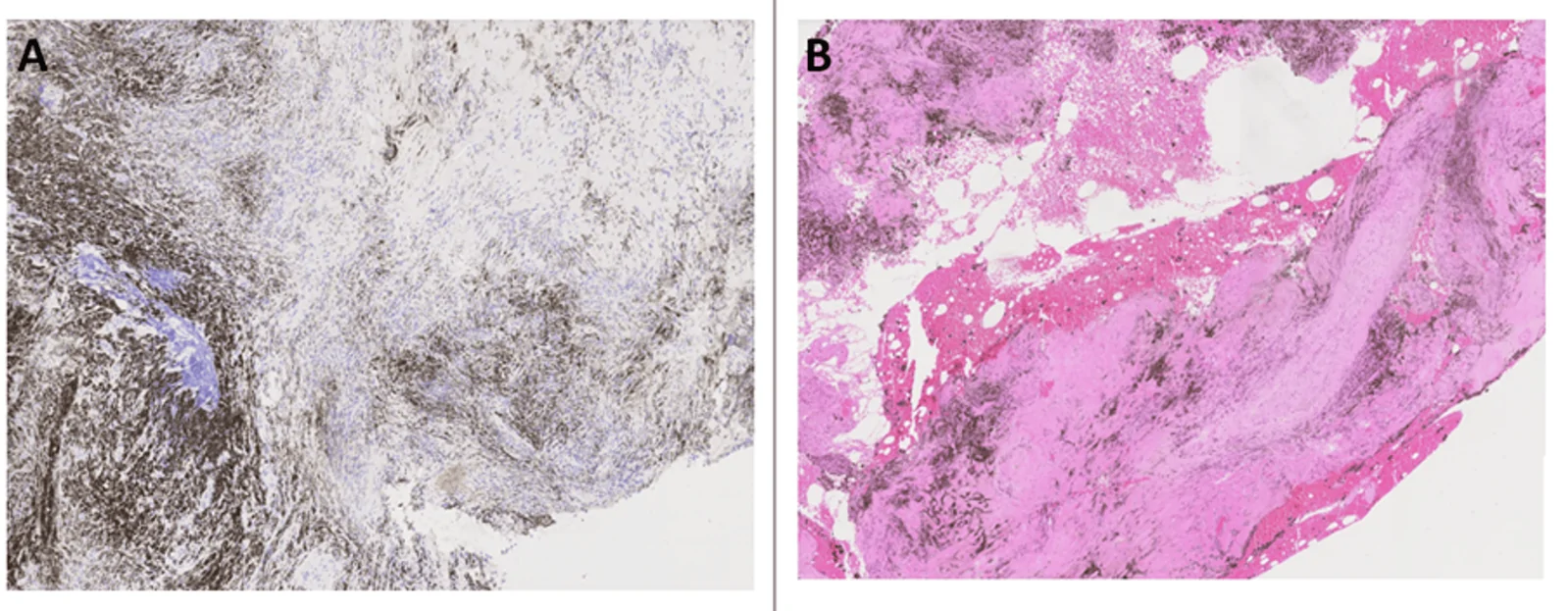
Extensive deposition of anthracotic pigment without any evidence of granulomatous, lymphoproliferative, and/or malignant disease (H&E, original magnification ×20) H&E: hematoxylin and eosin

However, because high radiological suspicion of malignancy persisted and the cytological diagnosis remained inconclusive, surgical mediastinoscopy was performed following multidisciplinary team (MDT) review. Histopathological examination of station 4R and station 7 lymph node biopsies demonstrated extensive deposition of anthracotic pigment without any evidence of granulomatous, lymphoproliferative, and/or malignant disease, confirming a diagnosis of anthracotic lymphadenitis (Figure 4A, 4B). The patient remained clinically stable and was managed conservatively.

## Discussion

This case illustrates a critical diagnostic pitfall: FDG-avid mediastinal lymphadenopathy is not pathognomonic for malignancy. Anthracotic lymphadenitis is an increasingly recognized cause of intense PET positivity and often exhibits high standardized uptake values (SUVs) despite only modest nodal enlargement [1,8]. The proposed mechanism involves retained, non-degradable carbonaceous microparticles providing persistent antigenic stimulation to nodal histiocytes and inducing a sustained macrophage-driven inflammatory response that manifests as hypermetabolism on PET-CT [5]. A retrospective series has demonstrated that benign anthracotic lymphadenitis can convincingly mimic metastatic disease, reinforcing that elevated SUV alone cannot differentiate benign inflammatory processes from nodal metastasis [4,9].

A thorough environmental exposure history provided the crucial diagnostic clue in this case, as biomass fuel smoke remains a major global source of indoor air pollution and is strongly linked to the development of pulmonary and nodal anthracosis [7]. The diagnostic differential becomes particularly challenging in patients with background risks for tuberculosis, as granulomatous infection and anthracosis frequently coexist or share overlapping radiological features, which require careful discrimination [1,10-15].

Although EBUS-TBNA is the preferred initial minimally invasive modality for mediastinal nodal evaluation, its diagnostic yield in anthracotic disease can be limited [10,16]. Cytology showing pigment-laden macrophages supports anthracosis; however, it may not conclusively rule out underlying malignancy, particularly when high radiological suspicion persists [10,16].

In our patient, the presence of pigmented macrophages on EBUS-TBNA was suggestive, but persistent clinical-radiological discordance necessitated surgical mediastinoscopy to obtain definitive histopathology. Ultimately, this case highlights that PET findings must always be interpreted in clinical context. In patients with relevant environmental exposure, anthracotic lymphadenitis should remain a key differential diagnosis, and full tissue confirmation remains mandatory before assuming advanced malignancy or altering definitive oncological management.

## Conclusions

Anthracotic lymphadenitis is an important and underrecognized benign cause of intense FDG-avid mediastinal lymphadenopathy that can convincingly mimic thoracic malignancy. In patients with a relevant history of inhalational or biomass fuel exposure, PET-CT findings must be interpreted with caution. When minimally invasive sampling via EBUS-TBNA yields non-definitive cytology, a surgical mediastinoscopy remains essential to obtain histological clarity and prevent inappropriate cancer staging and misdirected treatment.

## References

[1] Chang JM, Lee HJ, Goo JM, Lee HY, Lee JJ, Chung JK, Im JG (2006). False positive and false negative FDG-PET scans in various thoracic diseases. Korean J Radiol.

[2] Goo JM, Im JG, Do KH, Yeo JS, Seo JB, Kim HY, Chung JK (2000). Pulmonary tuberculoma evaluated by means of FDG PET: findings in 10 cases. Radiology.

[3] Kostakoglu L, Agress H Jr, Goldsmith SJ (2003). Clinical role of FDG PET in evaluation of cancer patients. Radiographics.

[4] Ivanick NM, Shrestha P, Podolsky MJ, Walavalkar V, Lucas CH, Gesthalter YB, Seeley EJ (2021). A retrospective observational study of benign anthracotic lymphadenitis and its association with PET avid lymph nodes in patients undergoing cancer evaluation. J Thorac Dis.

[5] Gupta A, Shah A (2011). Bronchial anthracofibrosis: an emerging pulmonary disease due to biomass fuel exposure. Int J Tuberc Lung Dis.

[6] Jamaati H, Sharifi A, Mirenayat MS (2017). What do we know about anthracofibrosis? A literature review. Tanaffos.

[7] Zhi X, Chen J, Xie F, Sun J, Herth FJ (2021). Diagnostic value of endobronchial ultrasound image features: a specialized review. Endosc Ultrasound.

[8] Giri S, Kumar L, Mane K, Uppin MS, Bhrugumalla S, Sundaram S (2024). 18F-fluorodeoxyglucose-avid benign anthracotic mediastinal lymphadenitis mimicking metastatic lymphadenopathy. Cureus.

[9] Park YS, Lee J, Pang JC (2013). Clinical implication of microscopic anthracotic pigment in mediastinal staging of non-small cell lung cancer by endobronchial ultrasound-guided transbronchial needle aspiration. J Korean Med Sci.

[10] Bruce N, Perez-Padilla R, Albalak R (2000). Indoor air pollution in developing countries: a major environmental and public health challenge. Bull World Health Organ.

[11] (2020). Evidence-based positron emission tomography: summary of recent meta-analyses on PET. https://link.springer.com/book/10.1007/978-3-030-47701-1.

[12] Hewitt RJ, Wright C, Adeboyeku D (2013). Primary nodal anthracosis identified by EBUS-TBNA as a cause of FDG PET/CT positive mediastinal lymphadenopathy. Respir Med Case Rep.

[13] Bilici A, Erdem T, Boysan SN, Acbay O, Oz B, Besirli K, Gundogdu S (2003). A case of anthracosis presenting with mediastinal lymph nodes mimicking tuberculous lymphadenitis or malignancy. Eur J Intern Med.

[14] Onitilo AA, Engel JM, Tanimu SB, Nguyen TC (2010). Anthracosis and large mediastinal mass in a patient with healed pulmonary tuberculosis. Clin Med Res.

[15] Lin WY, Hsu WH, Lin KH, Wang SJ (2012). Role of preoperative PET-CT in assessing mediastinal and hilar lymph node status in early stage lung cancer. J Chin Med Assoc.

[16] Silvestri GA, Gonzalez AV, Jantz MA (2013). Methods for staging non-small cell lung cancer: diagnosis and management of lung cancer, 3rd ed: American College of Chest Physicians evidence-based clinical practice guidelines. Chest.

